# Dietary **ω**-3 Polyunsaturated Fatty Acid DHA: A Potential Adjuvant in the Treatment of Cancer

**DOI:** 10.1155/2013/310186

**Published:** 2013-05-23

**Authors:** Nicolò Merendino, Lara Costantini, Laura Manzi, Romina Molinari, Donatella D'Eliseo, Francesca Velotti

**Affiliations:** Tuscia University, Department of Ecological and Biological Sciences (DEB), Largo dell'Università, 01100 Viterbo, Italy

## Abstract

**ω**-3 Polyunsaturated fatty acids (PUFAs), mainly present in fish oil, are part of the human diet. Among PUFAs, docosahexaenoic acid (DHA) has received particular attention for its anti-inflammatory, antiproliferative, proapoptotic, antiangiogenetic, anti-invasion, and antimetastatic properties. These data suggest that DHA can exert antitumor activity potentially representing an effective adjuvant in cancer chemotherapy. This review is focused on current knowledge supporting the potential use of DHA for the enhancement of the efficacy of anticancer treatments in relation to its ability to enhance the uptake of anticancer drugs, regulate the oxidative status of tumor cells, and inhibit tumor cell invasion and metastasis.

## 1. Introduction

Dietary fish oil (FO) has been shown to have beneficial effects on some chronic degenerative diseases such as cardiovascular disease [1, 2], rheumatoid arthritis [3], diabetes [4], other autoimmune diseases [5, 6], and cancer [7, 8]. The beneficial effects of FO seem to be due to its high content of the *ω*-3 polyunsaturated fatty acids (PUFAs) such as docosahexaenoic acid (DHA) and eicosapentaenoic acid (EPA). EPA is a long-chain *ω*-3 PUFA that has 20 carbon atoms and 5 double bonds (20 : 5); DHA has a longer chain, 22 carbon atoms and 6 double bounds (22 : 6). Both agents are essential fatty acids (FAs) that cannot be synthesized by mammals and thus must be obtained from dietary sources. 

DHA and EPA, as well as the other FAs, once ingested, can be uptaken and undergo to both cell passive translocation and carrier-mediated transmembrane translocation that involves various membrane-associated proteins [9]. Upon incorporation in cell membranes, PUFAs can be found either as constituents of membrane phospholipids (i.e., esterified FAs) or free molecules (i.e., free FA-FFA). In both forms, PUFAs give a substantial contribution to the physical properties of biological membranes, including membrane organization, ion permeability, elasticity, and eicosanoid formation [10, 11]. Taking into account these considerations, dietary DHA and EPA were established as significant nutrients involved in metabolic regulation. Moreover, some researches have established the capability of EPA and in particular of DHA to influence cancer proliferation [12], apoptosis [12, 13] and differentiation [12], as well as, to inhibit angiogenesis [14], tumor cell invasion [15] and metastasis [16]. These data suggest that DHA can both exert antitumor activity potentially representing an effective adjuvant in cancer chemotherapy and ameliorate some of the secondary complications associated with cancer, like cachexia [17, 18].

Despite progress made in recent years in cancer chemotherapy, advanced solid tumors, including advanced carcinomas, sarcomas, melanoma, and glioblastomas, still pose major difficulties in their treatment, and the traditional therapeutic modalities alone have not provided satisfactory long-term clinical results [19, 20]. Indeed, after one or several distinct lines of chemotherapy, in most cases only partial responses are obtained, meaning that after an initial pyrrhic success, tumors will resume growth, select therapy-resistant variants, and seal the patient's fate [21]. Even in those cases in which the tumor has apparently been removed completely (complete remission), micrometastases of dormant tumor cells (or cancer stem cells) often lead to relapse and to final therapeutic failure. Therefore, given the complexity of escape and survival to cancer development, most oncologists have reached the idea that no single therapy is sufficient to treat cancer [22, 23]. 

Evidence exists on the efficacy of DHA as anticancer adjuvant, with particular emphasis to its capability both to enhance the uptake of anticancer drugs, especially in cells otherwise resistant to these drugs, and to increase the pro-oxidant and proapoptotic efficacy of some chemotherapies [17]. This review focuses on the investigations on the potential use of DHA as adjuvant to improve the efficacy of anticancer treatment, acting at multiple levels such as the regulation of the oxidative status of tumor cells and the inhibition of tumor cell invasion and metastasis.

## 2. DHA as Adjuvant to Improve Tumor Cell Cytotoxicity Induced by Lipid Peroxidation and Oxidative Stress

### 2.1. Redox Status Management in Tumor Cells

As a consequence of aerobic metabolism, aerobic organisms produce a wide range of oxygen radicals and other reactive oxygen species (ROS), including free radicals (e.g., O_2_^•−^ and hydroxyl radical OH^•−^) and nonradical compounds (e.g., H_2_O_2_). ROS and reactive nitrogen species (RNS) are constantly generated inside cells by dedicated enzyme complexes (like NADPH oxidase and nitric oxide synthases) or as by-products of oxidation-reduction reactions, including those arising from mitochondrial respiration [24]. While some of these intermediates are useful against pathogens in the context of innate immunity, most are harmful to cells because they irreversibly damage proteins, lipids and nucleic acids and positively contribute, in different ways, to carcinogenesis and to malignant progression of tumor cells. 

When normal cells become cancerous, they exhibit elevated levels of endogenous ROS principally due to the accelerated metabolism needed to maintain the high proliferation rate typical of cancer cells [25]. Moreover, several studies indicate that high levels of mitochondrial ROS generation are essential for cellular proliferation and tumorigenesis. ROS can affect target gene expression through phosphorylation, activation and oxidation of transcription factors such as APEX1, NF-kB, p53 and HIF-1*α* [26–28]. Moreover, ROS can oxidize and inhibit signaling molecules such as p38 mitogen-activated protein kinase (MAPK) phosphatase, resulting in enhanced proliferation and survival of cancer cells [29]. 

High ROS levels imply that tumor cells also need to defend themselves from oxidative damage in order to survive and successfully spread. For example, the transforming activity of some oncogenes has been linked to their capacity to maintain elevated intracellular levels of reduced glutathione (GSH) (the principal redox buffer) [30] and high levels of antioxidant enzymes like superoxide dismutase 1 (SOD1) [31, 32]. Moreover, the “Warburg effect” adopted by cancer cells leads to activation of glucose metabolism and inhibition of mitochondrial respiration (i.e., cell catabolic processes that provides the highest quantity of reactive species), constituting thus a mechanism of protection that rescues tumor cells from oxidative stress and allows them to continue to proliferate [33]. On the other hand, ROS have been proposed as common mediators of apoptosis. Indeed, the vast majority of cytotoxic anticancer agents (including ionizing radiation, most chemotherapeutic agents, and some targeted therapies) work through the generation of ROS either directly or indirectly [34]. In fact, the accumulation of intracellular ROS causes the disruption of the mitochondrial membrane potential, the release of cytochrome c with consequent activation of the caspase cascade and ultimately cancer cell's demise through tumor cell death for apoptosis [35]. However, although various anticancer drugs initially induce ROS production, in most cancer cells the prolonged treatment with these drugs reduces the levels of ROS, resulting in drug-resistance. For example, it has been observed that cisplatin or chlorambucil initially induces ROS production in ovarian carcinoma A2780 cells, whereas prolonged drug treatments reduce ROS levels making those cells resistant to chemotherapy [36, 37]. Thus, a decrease of ROS level in prolonged drug-treated cells is not a secondary cellular outcome, but a primary mechanism of drug-resistance. Taking into account these considerations, “drug-combination” therapies might represent a good strategy to increase the efficacy of conventional anticancer treatments by acting as follows: (i) maintaining higher ROS levels in cancer cells, thereby precluding drug resistance; (ii) reducing cancer endogenous antioxidant defenses; and (iii) increasing drug uptake and thus apoptosis.

### 2.2. DHA as Adjuvant in the Modulation of the Redox Status in Tumor Cells

As mentioned above, in a variety of cancer types different anticancer chemotherapeutic agents have been shown to be more therapeutically effective when marine n-3 long-chain PUFAs were added to the diet [38]. The specific mechanisms behind these effects have not fully elucidated yet, but many scientific researches suggest that n-3 PUFAs may act at several levels. 

One of the main characteristics of PUFAs is the fact that they are highly susceptible to oxidation. Indeed, methylene group, located between two double bonds (–CH=CH–CH_2_–CH=CH–), is particularly vulnerable to radical attack by reactive species which entails the abstraction of hydrogen [39]. Among PUFAs, DHA having five of these methylene groups, is more susceptible to nonenzymatic lipid peroxidation [40]. DHA nonenzymatic oxidation is initiated after free radical attack (e.g., ^−^O_2_, H_2_O_2_), resulting in an unstable DHA-radical state that quickly undergoes isomerization and rearrangement of double bonds. These changes lead to the formation of conjugated dienes, which successively, after a further oxidation, are converted into lipid hydroperoxides. At this step, lipid hydroperoxides can follow two different ways: one leads to disintegration of the carbon chain and makes alkoxy radicals such as aldehydes (e.g., malondialdehyde, MDA) and alkanes and/or alkenes as byproducts (e.g., pentane); the other keeps the carbon chain intact, with the formation of peroxy radicals like isoprostane, isofuran and mono- or dihydroxy FAs [41]. The lipid peroxidation products, such as MDA, are highly toxic molecules for the cell, that are able to interact with nucleic acid bases to form several different adducts [42]. Moreover, the nonenzymatic lipid peroxidation into membranes triggers a further increase of formation of radical species in the cell. Therefore, as described above, considering that cancer cells contain a higher rate of reactive species compared to normal cells, the presence of DHA can lead to a high level of nonenzymatic lipid peroxidation into membranes, that keeps consistently high levels of ROS in the cells. Hossain et al. showed that DHA dose-dependently stimulated reactive species production and hence membrane lipid peroxidation, in HT-29 and Caco-2 colon carcinoma cells [43]. Moreover, other works show that this effect was enhanced when DHA was given in combination with some chemotherapeutic agents. In a report by Guffy et al., it has been shown that *in vitro* administration of DHA improved adryamicin uptake, cytotoxicity towards L1210 murine leukemia cells and increased tumor cell lipid peroxidation and oxidative damage [44]. Other studies have highlighted similar activities of DHA in combination with vincristine (VCT) chemotherapeutic agent. Indeed, DHA was able to increase VCT influx and cytotoxicity against both the KB-3-1 human cervical carcinoma cell line and the KB-ChR-8-5 VCT resistant cell variants. Similar results were observed in the NCG human neuroblastoma cell line and in NCG/VCR1 vincristine-resistant cells, where DHA enhanced the vincristine sensibility, by the enhancement of drug uptake and lipid peroxidation [45, 46]. These observations suggest that DHA may be capable of increasing the uptake of anticancer drugs in both resistant and sensible cells and that the incorporated DHA-mediated lipid peroxidation may lead to a greater cytotoxic effect compared to chemotherapy alone. However, the increased drug uptake by DHA was not seen in all tumor cell models. In fact, although it has been demonstrated that DHA is able to increase doxorubicin uptake in both P388 and P388/DOX (doxorubicin resistant) mouse leukemia cells [47] and in MDA-MB-231 and MCF-7dox (doxorubicin-resistant cells) human breast carcinoma cell lines, the same action has not been observed in parental MCF-7 human breast carcinoma cells [48]. This discrepancy might be due to the fact that different cell lines might have different cellular characteristics, such as membrane formation and configuration. However, even if DHA does not act in some cells as a drug uptake inducer, it can make cells more vulnerable to oxidative damage induced by exogenous agents. Thus, in some cases, DHA can make possible to overcome the maximum threshold of ROS tolerability by cancer cells, bypassing thus the oxidative stress resistance [49]. 

As above mentioned, cancer cells are capable of increasing their resistance to oxidative stress by increasing their endogenous antioxidant defenses. On this point, in our laboratory we have highlighted the ability of DHA to induce active GSH extrusion in the PaCa-44 pancreatic cell line [50]. We observed that intracellular GSH was dramatically reduced (more than 60%) by an active extrusion process after 6 h of cell treatment with DHA and that, in the presence of two specific inhibitors of carried-mediated GSH extrusion (such as cystathionine and methionine), DHA-induced GSH-extrusion process was reversed. Moreover, Ding and Lind showed that DHA treatment induced a 50% reduction of glutathione peroxidase-4 (GPx-4) protein expression and cytotoxicity in human ovarian cancer cell lines. Moreover, DHA-mediated cytotoxic effect was reversed by pre-treatment with vitamin E, suggesting that GPx-4 downregulation was due to oxidative stress [51]. Similarly, downregulation of SOD1 has been found in DHL-4 lymphoid cell line treated with DHA [31]. In the manuscript by Vibet et al., DHA-doxorubicin co-treatment caused an increase of ROS levels and a concomitant decrease of cytosolic GPx1 activity. This effect was detected both in MDA-MB-231 breast cancer cell line* in vitro* and in rat mammary tumors *in vivo* [52]. Furthermore, among the endogenous defenses to ROS, the Warburg effect (above mentioned) is another metabolic pathway adopted by cancer cells. In a recent paper, we have demonstrated that although the metabolism of human PaCa-44 pancreatic cancer cell lines mainly leans on glycolytic pathways (thus implementing the Warburg effect), after DHA treatment there was an overexpression of Kreb's cycle enzymes, indicating that cancer cell metabolism was switched in the Kreb's cycle activation [53].

As commented above, it has become clear the role played by the apoptotic alterations occurring during the development of neoplastic diseases. Thus, researches for possible therapeutic strategies involving the modulation of the apoptotic pathways have attracted considerable interest in the past few years. Oxidative stress has a fundamental role in apoptosis induction and many chemotherapeutic agents work generating directly or indirectly ROS, which lead to the key step in blocking cell cycle and apoptosis induction. Apoptosis occurs mostly through two mechanisms: the intrinsic and the extrinsic pathways. The first is caused by the disruption of mitochondrial membranes, the release into the cytoplasmic compartment of the mitochondrial cytochrome c, which in turn binds to the cytoplasmic apoptotic protease-activating factor (APAF) complex, triggering at first the activation of the initiator caspase-9 and then the executor caspases-3, -6, -7. The second pathway is initiated by the activation of death receptors by ligands like FasL, followed by the assembly of DISC (Death Inducing Signaling Complex), which, hydrolyzing the procaspase-8 or -10, causes caspase-8 or -10 activation. Then, caspase-8 or -10 activation results in the activation of executor caspases, which will be the real effectors of apoptosis [54]. Several evidence in the literature suggest the proapoptotic role of DHA, either alone or in combination of anticancer chemotherapies [17]. The apoptotic effect of DHA appears to take place through both the intrinsic and extrinsic pathways [13]. This role is further emphasized by the fact that DHA seems to be a potent inducer of apoptosis only for cancer cells and not for normal cells. For instance, it acts as a proapoptotic factor in colon cancer cells, whereas no significant proapoptotic effect was observed in the NCM460 normal human colon mucosal epithelial cell line [55], as well as cytotoxic effects were not observed in normal skin fibroblasts, microvascular endothelial cells and peripheral blood mononuclear cells derived from healthy donors [56]. These observations can be explained by the fact that normal cells might produce enhanced amounts of cytoprotective molecules such as lipoxins, resolvins and protectins in contrast to tumor cells which produce cytotoxic lipid hydroperoxides and other peroxides (as mentioned above) [57]. It has been demonstrated that the proapoptotic action of DHA is carried out by different ways, including the induction of lipid peroxidation and oxidative stress. DHA can be rapidly incorporated in mitochondrial membranes, altering their permeability and decreasing the mitochondrial membrane potential (MMP) [58, 59]. It has been also reported that DHA is mostly present in the mitochondrion in association with cardiolipins [60]. Cardiolipin-DHA molecules are under attack of radical species (highly presents in cancer cells) with the consequent decrease of their binding affinity for cytochrome c. Enhancement of their release as well as of other proapoptotic factors (e.g., the apoptosis-inducing factor, Smac/Diablo, Omi/HtrA2, and endonuclease G) from mitochondria to cytosol, leads to the induction of the activation of intrinsic apoptosis [61]. Sturlan et al. showed that DHA enhanced arsenic-trioxide-induced apoptosis in the arsenic-trioxide resistant HL-60 (myeloid leukemia), SH-1 (hairy cell-leukemia), and Daudi (Burkitt-lymphoma) cell lines and this effect was due to an increase of lipid peroxidation and a reduction of the mitochondrial membrane potential. Moreover, the authors showed that these effects were reversed by the addition of vitamin E [62]. Similar effects have been found in DHA-combined treatment with 5-fluorouracil (5-FU), oxaliplatin (OX) and irinotecan (IRI) in HT-29 human colorectal adenocarcinoma cells. The anticancer action of DHA, observed in presence of low doses of chemotherapeutic drugs (1 *μ*M 5-FU, 1 *μ*M OX and 10 *μ*M IRI), was carried out first by loss of mitochondrial membrane potential and then by caspase-9 activation [63]. 

## 3. DHA as Adjuvant to Improve the Antimetastatic Efficacy of Anticancer Therapies 

 Tumor metastasis is the primary cause of mortality in most cancer patients and thus the most life-threatening aspect of different types of tumors. Conventional chemotherapeutics exert cytotoxic activity against tumor cells affecting thus tumor growth. Therefore, chemotherapeutic effects on survival of cancer patients are generally interpreted as the consequence of their control on tumor cell growth, which in turn decreases tumor systemic spread. However, the initial chemotherapeutic efficacy is often hampered by the development by tumor cells of mechanisms of escape from the chemotherapeutic control, allowing residual cancer (stem) cells to growth, invade and metastasize. Therefore, the investigation on possible adjuvants in anticancer treatment affecting tumor cell invasion and metastasization is crucial to improve long-term therapeutic success of conventional anticancer agents, namely to decrease the mortality rate for cancer disease. 

 There is evidence that the intake of *ω*-3 PUFAs and in particular DHA inhibits not only the initiation of many kinds of tumors but also their progression, in that it inhibits metastases of murine and human tumors *in vivo* [64–67]. Indeed, marine fatty acid (*ω*-3 PUFA DHA and EPA) intake is associated with reduced all-case mortality in breast cancer patients [68]. In addition, there is some evidence that DHA increase the antimetastatic effect of anticancer drugs or other chemical compounds [16, 69, 70].

 Although the metastatic process is very complex and its molecular knowledge is still very limited, metastasization can be described as a sequence of phases in which cancer cells leave the original tumor site and migrate to other parts of the body via blood and lymphatic vessels [71]. The initial phase of the metastatic process results in the invasion by tumor cells of the surrounding primary tissue and the basement membrane. The invasion phase includes the following steps: (i) the detachment of tumor cells from surrounding primary cancer cells (i.e., loss of cell-to-cell-adhesion through downregulation of E-cadherin expression), (ii) the increased cell capacity to interact with extracellular matrix (ECM) proteins through adhesion molecules (e.g., integrins, cadherins, CD44), (iii) the degradation and remodeling of ECM by the secretion of hydrolytic enzymes (e.g., matrix metalloproteases-MMPs), and (iv) the migration through the degraded ECM (via mitogen-activated protein kinases-MAPKs, complex cascade of cytoskeleton rearrangement, cytokines and chemokines) towards blood and lymphatic vessels [71–73]. The second critical phase of the metastatic process includes the intravasation of tumor cells into blood or lymphatics, tumor cell survival and transport in the blood stream or the lymphatic system, followed by the arrest and extravasation at a distal site. Invasion, intravasation and extravasation underlie the tumor cell dissemination process that is often indicated in carcinomas as epithelial to mesenchymal transition (EMT) [73, 74]. Of note, EMT also occurs during normal embryonic development as precursor cells migrate along directions dictated by morphogenetic gradients [75]. Thus, EMT confers embryonic stem cell-like properties to tumor cells. Finally, the full accomplishment of the metastatic program requires that cells at a distant site survive and grow into secondary tumor masses. For clonal outgrowth at metastatic sites, as for final developmental cellular differentiation, EMT reversion such as mesenchymal to epithelial transition (MET) is required [76, 77]. This metastatic final step involves the capacity of tumor cells to proliferate, stimulate angiogenesis, and crosstalk with the component of the new microenvironment including parenchymal, stromal, and inflammatory cells [78]. The microenvironment surrounding both the primary tumor and metastases is regarded to be a prominent regulator of the metastatic potential [78]. In fact, stromal interactions contribute to invasion at the primary tumor site, where newly formed leaky blood vessels facilitate cancer cell intravasation; growth factors and cytokines produced by stromal cells (like tumor-associate macrophages) stimulate in trans-EMT [79, 80]. On the other hand, tumor cell-microenvironment interaction also involves the secondary tumor site, with promotion of neoangiogenesis, cell proliferation and crosstalk with pro-tumorigenic inflammatory immune cells and molecules [79–81].

 In this second part of the review, we first report some key evidence supporting the inhibitory effect of DHA on tumor metastasis and then we focus our attention on the influence of DHA on the initial phase of the metastatic process, namely the invasion phase. We also illustrate data supporting the idea that DHA might be combined with anticancer therapeutic strategies as adjuvant to improve their efficacy against tumor metastasization, thereby cancer patient survival extension.

### 3.1. DHA and Tumor Metastasis

 As early as 1994, Rose et al. started a certain number of studies on the effects of dietary fish oil *ω*-3 PUFAs, including DHA, on the growth and metastases of MDA-MB-435 human breast cancer cells in female nude mice [82]. Animals were fed with three isocaloric diets containing 23% total fat but different proportion of corn oil (rich in linoleic acid-LA) and menhaden oil (rich in EPA and DHA) [65, 82]. The authors reported that, in contrast to mice fed with diets rich in LA, mice receiving diets supplemented with EPA or DHA showed a significant suppression of both primary tumor growth rate and lung metastasis occurrence and severity, suggesting a role for EPA and DHA in the inhibition not only of tumor growth but also of metastasization of human breast cancer cells [82, 83]. Successively, the same authors investigated tumor responses to EPA or DHA administered immediately after surgical excision of the primary tumor (“postoperative adjuvant” activity). They found that DHA, but not EPA, significantly reduced lung involvement following the postexcision [84]. Therefore, based on these results, the authors suggested that PUFAs and in particular DHA may have a place in nutritional therapy of breast cancer as part of both neoadjuvant as well as postoperative adjuvant antimetastatic regimen. Similar results were obtained by Kinoshita et al., using purified DHA in a murine mammary metastatic tumor model [85]. Indeed, the authors observed that DHA suppressed not only the growth but also metastases of the MM48 murine mammary tumor transplanted into C3H/He mice. Of note, more recently, using a mouse model of MDA-MB-231 human breast cancer cell metastasis to bone, Mandal et al. showed that dietary fish oil DHA and EPA prevented the formation of osteolytic lesions in bone, suggesting a novel health effect of DHA or EPA on breast cancer cell metastasis to bone [86]. 

 The antimetastatic property of DHA was also investigated using different experimental models of colon cancer metastases. It has been shown that dietary marine oil (*ω*-3 PUFA EPA and DHA) inhibited the growth as well as the pulmonary colonization of a transplantable colon tumor (CT-26) implanted at the descending colon of male Balb/c mice [64]. Then, Iigo et al. and Suzuki et al., using the same subcutaneous implanted highly metastatic colon carcinoma 26 (Co 26Lu) model, found that a DHA-rich diet, when administered together with tumor cells, dramatically suppressed lung metastases (58% fewer colonies than control). Moreover, they found that *in vivo* DHA-treated tumor cells maintained their low potential for lung colony formation when transferred to new hosts, proposing that the effect of DHA was exerted directly on the metastatic ability of the tumor cell and not on the microenvironment [87, 88]. One further study, was performed on a model of colorectal metastasis in male rats (WAG/Rij) fed with a diet containing an EPA/DHA mixture (1.96% fish oil; EPA : DHA ratio 3 : 2) three days before and 28 days after splenic injection of CC531 cells (a moderately differentiated colon adenocarcinoma). A 70% reduction in incidence and 50% reduction in liver metastasis size as compared to control rats fed with 15% coconut oil was found [89]. Finally, Ichihara et al. reported the high therapeutic effects of intravenous injection of hybrid liposomes DMPC/DHA (composed of 50 mol% L-*α*-dimyristoylphosphatidylcholine-DMPC and 50 mol% DHA) on the hepatic metastasis mouse model of HCT116 human colon carcinoma cells. This effect was also associated with a prolonged murine survival [90]. 

 Gleissman et al. showed that DHA, given daily by gavage in atymic rats, delayed the progression of established aggressive human neuroblastoma xenografts [67].

 Finally, the antimetastatic activity of DHA was also observed by Yam et al. in the well-characterized model of Lewis Lung Carcinoma (3LL) metastases in C57BL/6J mice [66]. Interestingly, in the same experimental model, these authors also investigated the antimetastatic property of dietary fish oil (DHA and EPA) administered in combination with vitamins E and C and cisplatin [69]. Indeed, C57BL/6J mice bearing Lewis lung carcinoma (3LL) were fed ad libitum with one of the three isocaloric diets containing 5% soybean oil supplemented with 40 mg/kg *α*-tocopherol acetate (SO diet), or 4% fish oil plus 1% corn oil and basal amounts of vitamin E (FO diet) or FO diet supplemented with vitamins E and C (FO + E + C diet). These diets were tested in combination with the conventional cytotoxic agent cisplatin in a series of regimens and tumor growth and lung metastasis were monitored. Both the FO dietary groups showed significantly lower tumor development than the SO group in all examined parameters, indicating that *ω*-3 PUFAs exert anticancer activity. However, the FO diet, in comparison with the FO + E + C diet induced a significantly slower rate of tumor growth as well as lower metastatic load, as reflected in lung weight. The authors proposed that the decreased anticancer activity of FO by the addition of vitamins E and C could be explained by the decrease of oxidized *ω*-3 PUFAs, that, accumulated in the membranes and the cytosol of tumor cells, reduced their vitality and eventually lead to their death. Cisplatin treatment with the SO diet had no apparent therapeutic effect, while cisplatin combined with the FO diets significantly reduced the metastatic load. Therefore, these results suggest an adjuvant function of DHA and EPA in chemotherapy on spontaneous metastatic dissemination [69].

 Despite evidence from preclinical studies for antimetastatic activity of DHA, to the best of our knowledge no published studies have yet investigated the effect of DHA in patients with metastatic tumors. We identified only one published human study on the antimetastatic effect of combining DHA with a conventional chemotherapeutic regimen. On the basis of the ability of DHA to increase the efficacy of anticancer agents by induction of oxidative stress, a phase II study evaluated the addition of 1.8 g DHA daily to an anthracycline-based chemotherapy regimen for 25 patients with metastatic breast cancer. Patients were dichotomised into two groups based on high or low DHA incorporation into plasma phospholipids. The high DHA-incorporation group had a significantly longer time to disease progression (median 8.7 months versus 3.5 months) and overall survival (median 34 months versus 18 months). Although the small number of patients involved in the study does not allow a definitive conclusion on the efficacy of this combined treatment, the data indicate that adjuvant treatment with DHA may improve the outcome of chemotherapy, in terms of response rate, time to progression, and overall breast cancer survival [70]. 

 The molecular mechanisms by which DHA, alone or in combination with other agents, may affect the metastatic potential of tumors remain unclear. However, several molecular mechanisms have been proposed. In most studies above-mentioned, the investigations were focused on changes in the chemical content of tumor FAs and alterations of tumor membrane characteristics induced by the uptake of DHA associated to the displacement of arachidonic acid (AA) in phospholipid membranes of tumor cells [65, 66, 82, 85, 87, 91]. Incorporation of DHA in the tumor cell membrane results to some extent in a change in its lipid composition, which might make plasma membrane considerably less fluid and less deformable [92]. Thus, it has been proposed that the antimetastatic activity of DHA may be related to pronounced changes in the FA composition of tumor cells, which impair tumor cell membrane and decrease the ability to metastasize [82, 87]. Moreover, it has been observed that the increased representation of DHA in tumor phospholipids, associated with a statistically significant reduction in AA concentrations, suppresses AA-derived eicosanoid (PGs) biosynthesis thus decreasing prostaglandin (PG) E2 concentration [65, 66, 85]. This point may be crucial, since PGE2 production results in suppression of immune responses to cancer cells and in promotion of inflammation, as well as, enhancement of cell proliferation, neo-angiogenesis and invasion. Furthermore, Suzuki et al., in the metastatic colon carcinoma model, found that dietary DHA caused a decrease in metalloprotease-9 (MMP-9), which was well correlated with AA content in tumor tissues (*r* = 0.900, *P* < 0.001), suggesting that inhibition of metastasis by DHA might be due to depressed type-IV collagenase activity [88]. This is consistent with data in the literature reporting the influence of DHA on the one-carbon cycle, thereby contributing to increased homocysteine and oxidative stress leading to decrease gene expression of MMPs and to increase that of specific endogenous inhibitors of MMPs such as tissue inhibitors of metalloproteinases (TIMPs) [93]. Finally, Mandal et al., using the mouse model of human breast cancer cell metastasis to bone, found that fish oil supplemented with DHA and EPA significantly inhibited mRNA and protein levels of the cell-surface CD44 adhesion molecule (involved in cell-cell interactions, cell adhesion and migration) in the aggressive MDA-MB-231 tumors, thus identifying a novel DHA function in tumor cells that is the targeting of the cell-intrinsic pro-metastatic CD44 molecule expression [86]. This is a very interesting data, since the acquisition of increased expression of CD44 by noninvasive breast cancer cells correlates with the induction of EMT necessary for metastatic potential [94]. However, several other targets of DHA including cyclooxygenase-2 (COX-2), nuclear factor kappa-light-chain enhancer of activated B cells (NFkB), peroxisome proliferator-activated receptor-*γ* (PPAR-*γ*), mitogen-activated protein kinases (MAPK), Akt (also known as Protein Kinase B), and B-cell lymphoma/Bcl-2-associated X protein (BCL-2/BAX) play an important role in the suppression of metastases and excellent reviews are already available on their implication in DHA influence on tumor cell proliferation [8], angiogenesis [14] and immune system response [95], all critical events in the metastatic process. Therefore, in the next chapter we focus our attention on the influence of DHA on tumor cell invasion, the first phase of the metastatic process, illustrating studies that investigated the effect DHA on the invasion capability of murine and human cancer cells *in vitro*. 

### 3.2. DHA and Tumor Cell Invasion

 An early study on the effect of DHA on tumor cell invasion is dated 1993, when Connolly and Rose, using an *in vitro* invasion assay, examined the effect of LA, EPA, and DHA on the invasive capacity of the aggressive MDA-MB-435 human breast cancer cell line. They reported that although all these agents did not affect the migration of tumor cells through gelatin, EPA and DHA (at concentration of 0.25 and 0.5 *μ*g/mL, that did not inhibit cell growth), but not LA, significantly inhibited the invasion of tumor cells through Matrigel [96]. Recently, Altenburg and Siddiqui found that treatment of the aggressive MDBA-MB-231 breast cancer cells with *ω*-3 PUFAs resulted in reduced surface but not overall C-X-C chemokine receptor type 4 (CXCR4) expression and subsequently in reduced CXCR4-mediated cell migration. The authors also suggest that the possible mechanism behind the reduced CXCR4 activity may be the disruption of the lipid raft domains by PUFAs, which results in a partial displacement of CXCR4 [97]. According to the anti-invasion activity of DHA in aggressive breast cancer cell lines, Blanckaert et al. showed that the invasive phenotype of the MDA-MB-231 human breast carcinoma cell line was markedly decreased following cell incubation with 100 *μ*M of DHA for 24 h, whereas they could not observe any effect when cells were treated with 20 *μ*M of DHA whatever the incubation time, suggesting that high doses of DHA are required for this activity [98]. Other authors investigated the anti-invasion effect of combining DHA with another nutritional compound such as genistein [16]. Genistein, an isoflavonoid isolated from soybean, has been shown to possess anticancer activities and is a potent inhibitor for a number of tyrosine kinases. Horia and Watkins tested the combination of genistein and DHA for the synergistic inhibition of cell invasiveness, suppression of PGE2 production and COX-2 expression in MDA-MB-231 cancer cells. Their data demonstrated an additive effect of DHA and genistein in suppressing cell invasiveness and the endogenous production of PGE2. Furthermore, the combination of DHA and genistein did not enhance the suppression of COX-2 gene expression but appeared to work through peroxisome proliferator-activated receptor-c/pregnane X receptor-*α*- (PPARc-PXR*α*-) mediated pathways for reduced PGE2 production [16], thus suggesting alternative molecular targets for DHA to PGE2 inhibition.

 The anti-invasive activity of DHA was also investigated using other types of experimental tumor models. McCabe et al. demonstrated that DHA (10 *μ*M for 24 h, which had no effects on cell proliferation) significantly inhibited (48.48%) the invasion of caki-1 renal cell carcinoma cell line through Matrigel. They also reported that this effect was associated to increased tumor cell levels (17.42%) of TIMP-1, and that similar increased levels were found when PGE2 production was inhibited, suggesting that the reduction of the invasive profile is regulated by tumor PGE2 production levels [99]. In the 70W human melanoma cell line (that metatstazise to the brain in nude mice), Denkins et al. demonstrated that DHA (50 *μ*M for 24 h) decreased Matrigel invasion and that this effect was associated to the inhibition of the COX-2 expression, which in turn downregulated PGE2 production [100]. Moreover, Xia et al. examined the effect of alteration in the n-6/n-3 fatty acid ratio on the invasive potential of human lung cancer A549 cells. These cells had a marked reduction of the n-6/n-3 fatty acid ratio, because they were transfected with the Caenorhabditis elegans fat-1 gene, which encods an n-3 desaturase that converts n-6 to n-3 fatty acids. Cell adhesion assay showed a significant delayed adhesion and retarded colonization. Matrigel assay indicated a 2-fold reduction of cell invasion in the fat-1 transgenic cells when compared with the control cells. Microarray and quantitative polymerase chain reaction revealed a downregulation of several adhesion/invasion-related genes (MMP-1, integrin-*α*2 and nm23-H4) in the fat-1 transgenic cells, suggesting that the reduced invasion and colonization potential of human lung cancer cells induced by decreased n-6/n-3 fatty acid ratio were probably due to downregulation of cell adhesion/invasion-related molecules [101]. Furthermore, in U87 malignant glioma cells, Mita et al. showed that DHA inhibited and AA stimulated tumor cell migration. The authors also showed that the antimigratory effect by DHA was dependent on its binding with the brain fatty acid-binding protein (FABP7) which move to the nucleus and cooperates with DHA for the activation of the PPAR*γ* transcription factor, thereby downregulating the COX-2-PGE2 pro-migratory pathway. The authors thus proposed FABP7 and its fatty acid ligands as key therapeutic targets for controlling the dissemination of malignant glioma cells within the brain [102]. Very recently, Sun et al. also found that DHA (100 *μ*M for 24 h) inhibited migration as well as invasion of the Bel-7402 human hepatocellular carcinoma cell line and that those inhibitions paralleled MMP-9 decrease [103]. Finally, in our laboratory we recently showed that, in contrast to AA, DHA (25, 50, and 100 *μ*M for 24 h, that did not affect cell proliferation) inhibited in a dose-dependent manner the invasion of RT112 urinary bladder and PT45 pancreatic carcinoma cell lines through Matrigel. Moreover, we showed that, in contrast to AA, the inhibition of cancer cell invasion paralleled DHA-induced downmodulation of the tumor-expressed chymotrypsin-like serine protease granzyme B (GrB) [15]. Although GrB was originally known as a cyotoxic molecule of cytoplasmic granules of cytotoxic lymphocytes [104], it was recently also characterized for its extracellular functions [105], such as invasion promotion of cancer cells [106]. The inhibitory effect of DHA on GrB expression is consistent with results from Kun et al., showing that dietary *ω*-3 PUFAs inhibited the expression of GrB in a rat model of small bowel transplant chronic rejection [107]. We also demonstrated that GrB was expressed in cancer cells *in vitro* and *in vivo*. GrB was capable of degrading ECM components and promoting invasion of bladder and pancreatic cancer cells *in vitro*. Moreover, GrB tumor expression was significantly associated with tumor EMT* in vivo*, as well as, with the pathological tumor spreading [15, 107]. Taking into account these results, we proposed a possible causative role of GrB in the inhibition of bladder and pancreatic cancer cell invasion by DHA [15].

## 4. Conclusions

In conclusion, taking into consideration the data in the literature, it appears that DHA has the ability to inhibit metastasis in preclinical *in vivo* tumor models as well as invasion and migration in *in vitro* tumor cells. It also appears that a “combination therapy” of DHA and antitumor drugs may increase the cytotoxic efficacy of drug treatment alone, since it should allow cancer cells to maintain higher levels of ROS (thereby precluding drug resistance), reduce endogenous antioxidant tumor cell defenses, and increase drug uptake. Despite these encouraging results, there is still a need to verify whether DHA supplementation can improve the antimetastatic efficacy of chemotherapeutic and radiotherapeutic anticancer regimens in humans.
